# Assessment of retention force and bone apposition in two differently coated femoral stems after 6 months of loading in a goat model

**DOI:** 10.1186/s13018-014-0069-4

**Published:** 2014-08-16

**Authors:** Knut Harboe, Nils R Gjerdet, Einar Sudmann, Kari Indrekvam, Kjetil Søreide

**Affiliations:** 1 Department of Orthopaedic Surgery, Stavanger University Hospital, Stavanger 4068, Norway; 2 Department of Clinical Medicine, University of Bergen, Bergen, Norway; 3 Biomaterials, Department of Clinical Dentistry, University of Bergen, Bergen, Norway; 4 Kysthospitalet in Hagevik, Clinic of Orthopaedic Surgery, Haukeland University Hospital, Bergen, Norway; 5 Department of Gastrointestinal Surgery, Stavanger University Hospital, Stavanger, Norway

**Keywords:** Hydroxyapatite, Calcium phosphate, Loaded animal model, Hip replacement, Implant stability

## Abstract

**Background:**

Since the introduction of uncemented hip implants, there has been a search for the best surface coating to enhance bone apposition in order to improve retention. The surface coating of the different stems varies between products. The aim was to assess the retention force and bone adaption in two differently coated stems in a weight-bearing goat model.

**Materials and methods:**

Hydroxyapatite (HA) and electrochemically deposited calcium phosphate (CP; Bonit®) on geometrically comparable titanium-based femoral stems were implanted into 12 (CP group) and 35 (HA group) goats. The animal model included physiological loading of the implants for 6 months. The pull-out force of the stems was measured, and bone apposition was microscopically evaluated.

**Results:**

After exclusion criteria were applied, the number of available goats was 4 in the CP group and 11 in the HA group. The CP-coated stems had significantly lower retention forces compared with the HA-coated ones after 6 months (CP median 47 N, HA median 1,696 N, *p* = 0.003). Bone sections revealed a lower degree of bone apposition in the CP-coated stems, with more connective tissue in the bone/implant interface compared with the HA group.

**Conclusion:**

In this study, HA had better bone apposition and needed greater pull-out force in loaded implants. The application of CP on the loaded titanium surface to enhance the apposition of bone is questioned.

## Introduction

Total hip arthroplasty (THA) is one of the most frequently performed procedures in orthopaedic surgery with more than 7,000 THAs performed each year in Norway alone [1]. Cemented prostheses, hybrid implants (a cemented cup and an uncemented stem) and entirely uncemented implants are increasingly being used. Uncemented implants pose a higher risk for revision than cemented stems [2]–[4] and as such may represent additional health burden for patients. For uncemented stems, several factors have been attributed to stem failure and the need for revision, including an increasing rate of infection [5], aseptic loosening and recurrent dislocations [1],[6],[7]. To overcome the complications, several solutions of improvement have been suggested. Among these are the investigations into different geometric surface structures [8] or search for the optimal surface coating of stems. The aims of such investigation are to enhance bone apposition and improve the retention force and, consequently, the longevity of the implants [1],[9],[10].

The surface coatings of different stems usually include forms of hydroxyapatite to enhance the apposition of bone [1],[11]. A previous study reported on a novel stem design that was well fixed in a goat model [12]. The implant was made from titanium alloy with hydroxyapatite coating. The stem was designed to facilitate removal in the event of stem failure and the need for revision surgery. A previous version of the same stem design was made from the same titanium alloy but was surface coated with calcium phosphate. Thus, the aim of the current study was to evaluate the difference in retention force and morphology of tissue between femoral stems having two different resorbable coatings.

## Materials and methods

The study was performed on goats receiving hip implants and subjected to their natural environment involving physiological loading. The goats were separated into two different groups for evaluation, depending on the type of coated stem implants received: either the calcium phosphate (CP group) or the hydroxyapatite (HA group) coated stems. The novel stem design and animal care have previously been reported in detail [12], and the two groups were part of an innovative process to find the optimal design and surface treatment of the final implant.

**Figure 1:**
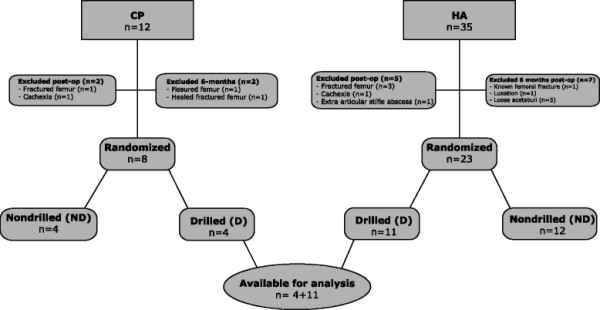
**Design of the study.** Design of the study comparing hydroxyapatite and calcium phosphate coating of an experimental hip stem in a goat model.

In the CP group (Figure 1), 12 adult female goats, weighing on average 50 (38–58) kg, were admitted to the Vivarium facility, University of Bergen, 2 weeks before surgery. This first study was a pilot with a limited number of goats. They were clinically examined and blood samples were analysed. They had been vaccinated against paratuberculosis (*Mycobacterium avium* subspecies *paratuberculosis*) as kids.

In the HA group, 35 adult female goats, weighing on average 50 (40–65) kg, were admitted to the Norwegian School of Veterinary Science, Section for Small Ruminant Research and Farm Animal Health, Sandnes, 4 months before surgery. The number of goats was increased as the funding after the pilot study had increased, and the new site was selected to allow this increased number. They were clinically examined and checked for intestinal and lung parasites by a veterinary surgeon, treated against parasites (Panacur, Intervet, Boxmeer, The Netherlands) and vaccinated against clostridial diseases (Ovivac P vet, Intervet, Boxmeer, The Netherlands). All were clinically healthy and none had signs of caprine arthritis encephalitis (CAE), which has a low prevalence in this area of Norway. Serological test for CAE was, however, not performed. They had all been vaccinated against paratuberculosis as kids.

The studies were approved by the Norwegian Animal Research Authority (Reference number 2006350 and 07/82783). The animals were euthanized if they luxated a hip, fractured a limb or developed an infection that did not respond to antibiotics within 14 days.

Anaesthesia was induced with xylazine (Rompun vet., Bayer, Berlin, Germany) 0.2 mg/kg i.m. and ketamine (Ketalar, Pfizer, New York, NY, USA) 11 mg/kg and maintained with isoflurane 4% and morphine 2 mg/kg i.v. every 2 h during surgery. A ventricular drain, coupled to a closed bag, was introduced into the womb to relieve gas produced there.

All goats received a total hip arthroplasty using uncemented femoral stems of own design, combined with a cemented canine acetabular cup and a modular head. The operative procedure, the basic stem coated with calcium phosphate and overall logistics were introduced in 2006 for the CP group. Later, stems with an updated stem design coated with hydroxyapatite were likewise inserted in 2008 (HA group).

**Figure 2:**
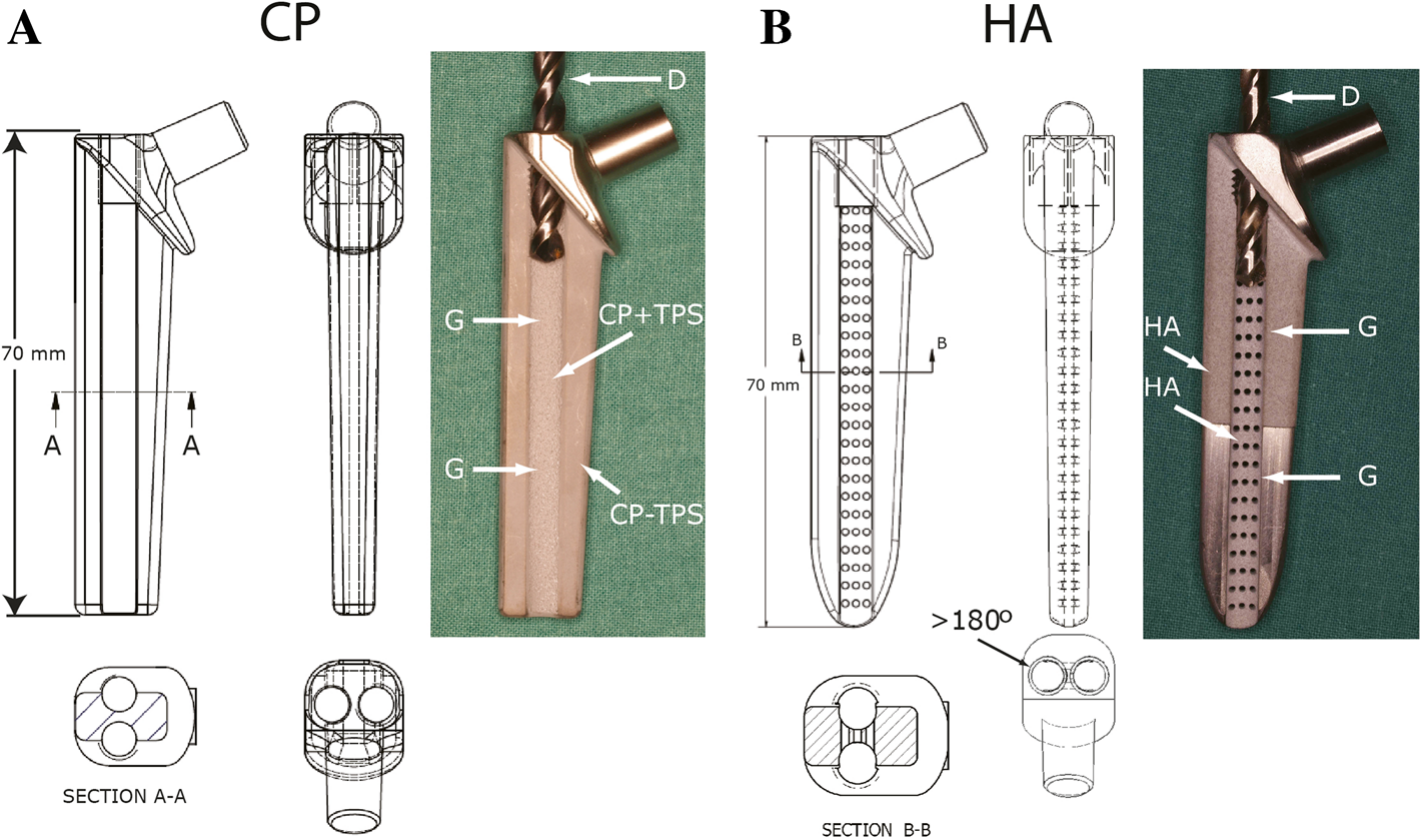
**Descriptive details and design of the study implants. (A)** Front, side and top views. Far right, stem with drill bit. *CP-TPS* calcium phosphate-coated area, *CP + TPS* calcium phosphate on porous commercially pure titanium in groove, *D* drill bit, *G* groove. **(B)** Front, side and top views. Note on top view that the grooves are more than 180° to prevent the drill bit from going astray. Far right, stem with drill bit. *HA* hydroxyapatite-coated area, *D* drill bit, *G* groove. Transverse canals are 1 mm in diameter. (Reproduced with permission from VCOT [12]).

Both groups received the same novel stem design [12]. Briefly, based on preoperative radiographs of goat's femora, a stem was produced with standard Computer Numerical Control (CNC) technology (Aarbakke Inc., Bryne, Norway) and made from titanium alloy (Ti6Al4V grade 5) in both groups. The stem in the CP group was one size, measuring 70 mm in length. The stem had two semicircular longitudinal grooves. The stems were grit blasted to a roughness (*R*_a_) of 1.5 μm, and porous commercially pure titanium (titanium plasma spray (TPS), DOT GmbH, Rostock, Germany) was added to the grooves by vacuum plasma spraying; porosity was 21% and pore size approximately 40 μm. The area outside the groove was not covered with TPS. The coated stem had no transverse canals from groove to groove. Finally, the whole stem beneath the collar was coated with calcium phosphate, with a thickness of 15–20 μm (Bonit®, DOT GmbH, Rostock, Germany) (Figure 2).

In the HA group, there were two stem widths to accommodate different femoral sizes in the goat population, both measuring 70 mm in length. The stems also had two semicircular longitudinal grooves, and these were connected with 69 transverse canals measuring 1 mm in diameter. The stems were grit blasted to a roughness (*R*_a_) of 3–7 μm in the proximal half and the full length of the grooves. Hydroxyapatite (Sulzer Metco F6264, Zurich, Switzerland) was applied all over the blasted area. The coating was approximately 80 μm thick and applied by plasma spraying (Sulzer Metco, Zurich, Switzerland) with a porosity of <5% and crystallinity of approximately 67% (Figure 2). This coating was chosen because of its properties similar to those of the coating of the Corail AMT stem (DePuy, Warsaw, IN, USA).

All stems were gamma sterilized and packed (Gamma Service GmbH, Dresden, Germany, in the CP group and Früh, Zurich, Switzerland, in the HA group) before use. In both groups, a standard 17-mm total hip arthroplasty head designed for canine use and a corresponding cemented socket (HDP, Biometrix, Boonton, NJ, USA) were used. Both groups where operated according to the procedure described previously [12].

Post-operative radiographs were taken before recovery from anaesthesia. A dose of atipamezole hydrochloride, average 0.75 mg (0–2 mg) (Antisedan Orion, Espoo, Finland), and cefalotin 1 g i.m. was given every 3 h for 9 h (Cefalotin, ACS Dobfar Generics, Luxembourg City, Luxembourg). To prevent hip dislocation, the animals were retained individually in a specially tailored support sling for three post-operative days allowing full weight bearing [13]. During this period, the animals received buprenorphine (Temgesic, Schering-Plough, Kenilworth, NJ, USA) 5 μg/kg s.c. twice a day for pain relief. The animals were kept in a separate stable with one or two other animals for an additional 2–3 days with no restrictions before joining other operated animals, which were kept in a common stable until transfer. In the CP group, the goats were transported to a local farm for the duration of the study period. The goats were kept indoors during the winter. In the HA group, 4 weeks after the last animal was operated on, they were all let free in an outdoor enclosed area with access to hills and shelter.

In the CP group, the gait of the goats was scored every 4 weeks combined with weighing. In the HA group, we scored the gait every 14 days and weighed every 4 weeks. In both studies, the goats were scored with a modified de Waal score [12] and clinically examined by a veterinarian when indicated.

The goats were euthanized with a standard bolt gun and exsanguination (CP group) or with a bolus of 2 g of pentobarbital intravenously (Pentobarbital, NAF, Ås, Norway) (HA group). The left femur and ipsilateral part of the pelvis were explanted with the prosthetic components *in situ*. The specimens were kept on ice until biomechanically tested the next morning.

All stems were allocated into two subgroups by coin toss: drilled (D) and non-drilled (ND), in both studies. All tissue in the grooves was removed by a 4.5-mm drill bit in the drilled subgroup. The studies were approved by the Norwegian Animal Research Authority (Reference number 2006350 and 07/82783).

The stems were pulled out by a servo-hydraulic universal test machine (MTS 810, Minneapolis, MN, USA) using a cross-head speed of 0.25 mm/s with continuous sampling of the load and displacement data. The specimens were fixed to the test machine by embedding the distal part of the femur in acrylic cement (Meliodent, Heraeus-Kulzer GmbH, Hanau, Germany) in a custom-made steel cylinder that allowed alignment. The stem was fixed to the cross-head by a rod that fitted threads in the prosthesis. The testing took place at room temperature, keeping the specimens moist throughout the procedure [14].

To test the effect of the bonding of the coatings to the bone, we compared the subgroups from both studies that had the longitudinal grooves on each side drilled (subgroup D). In this way, we minimize the difference of the anchoring of the groove to the surrounding bone (Figure 1).

After the biomechanical testing, the proximal part of all femurs (above the level of acrylic cement during the biomechanical testing) was immersed in 4% neutral buffered formalin. Bone samples from a proximal (stem collar), intermediate and distal (about 1 cm above the end of the stem) part were decalcinated with ethylenediaminetetraacetic acid (EDTA) demineralizing solution (1,000 ml 4% unbuffered formalin, 75 g EDTA, 14 g NaOH) and embedded in paraffin, cut into 2–3-μm sections and stained with hematoxylin, erythrosine, saffron (HES) according to the laboratory protocol.

Bone apposition was evaluated by light microscopy for bone growth and for the presence of immunological cells, which could indicate an adverse reaction to the coating.

SPSS 19.0 (SPSS Inc., Chicago, IL, USA) was used for data presentation and statistical analysis. Median and range were calculated for descriptive numbers. The two groups were compared with a non-parametric test (Mann–Whitney) with regard to pull-out force. The significance level was set at *p* < 0.05, two sided.

## Results

Of the 47 goats included in the overall project, a subgroup of 15 was available for analysis (Figure 1).

In the CP group (*n* = 12), one goat was euthanized due to perioperative femoral fracture 12 days post-operatively after being butted by another goat, and one was euthanized due to cachexia 5 weeks post-operatively. At 6 months, two goats were excluded due to a previous fracture that had healed and a fissure.

In the HA group (*n* = 35), three goats were euthanized due to perioperative femoral fracture, one died of cachexia 5 weeks post-operatively, and one was euthanized due to an extra-articular abscess at the stifle on the operated side 5 months post-operatively. Two goats managed to escape the sling but were repositioned after a short period.

In both studies, several of the goats went on butting after being set free from the slings, i.e. after 3 days. At autopsy 6 months post-operatively, we found one animal in the HA group with an unacknowledged luxated hip with a neoacetabulum above the cemented socket (this animal had previously escaped the support sling), one known healed reamed hole laterally in the femur, and five loose acetabuli. These goats were also excluded from the study (Figure 1).

After a temporary mean weight reduction for 4 weeks, there was a mean weight gain indicating that the goats thrived and acted normally in both groups (CP group: post-operative 49.1 kg, 4 weeks 49.0 kg, 6 months 53.0 kg; HA group: 51.3 kg, 48.2 kg, 55.4 kg, respectively).

In the CP group, the goats had a median gait score of 4 (range 3–5) after 4 weeks post-operatively and 4 (range 4–5) at the end of the experiment at 6 months. In the HA group, the goats had a median gait score of 4 (range 3–5) 4 weeks post-operatively and 5 (range 3–5) at the end of the experiment. There was no statistical difference in gait score between the CP group and the HA group in the drilled groups (*p* = 0.95).

**Figure 3:**
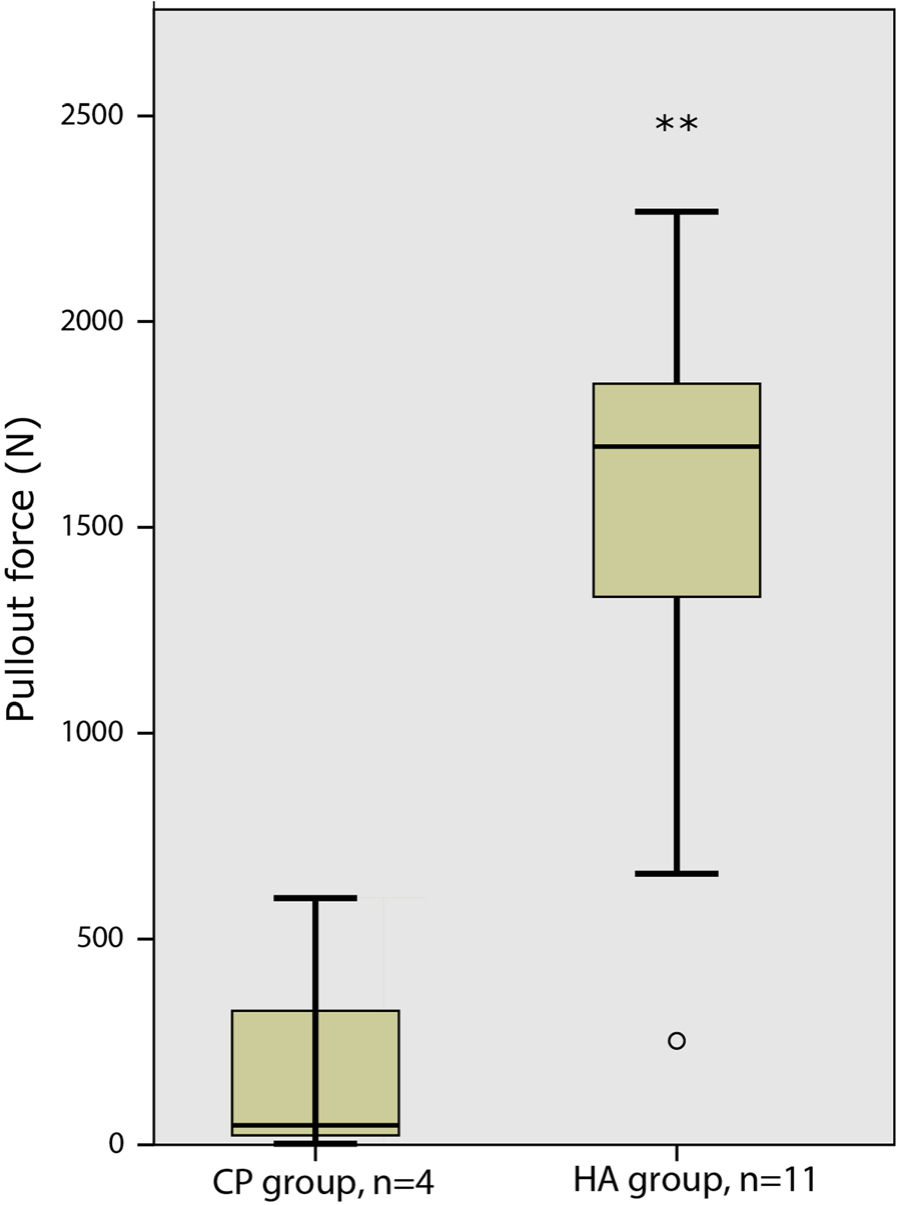
**Pull-out force of study implants.** Box-and-whiskers plot of pull-out force measured in CP and HA groups. The *box* indicates the 25th and 75th quartiles, with the median (*bold line*). The *whiskers* represent minimum and maximum values. An outlier is indicated by the *circle*. The difference in median was statistically significant (**p = 0.003).

All stems were retrieved with negligible waste of peri-prosthetic bone tissue. Stem pull-out resistance was statistically different between the drilled groups: CP group median 47 (range 3–598) N vs. HA group median 1,696 (252–2,267) N, *p* = 0.003 (Figure 3).

In the CP group, there was little macroscopically visible bone attached to the stem outside the groove. Visually, there were no remnants of calcium phosphate. In most stems of the HA group, bone had formed on the grit-blasted and hydroxyapatite-coated area and in most through holes in the grooves. The hydroxyapatite coating was only visible as occasional small patches.

**Figure 4:**
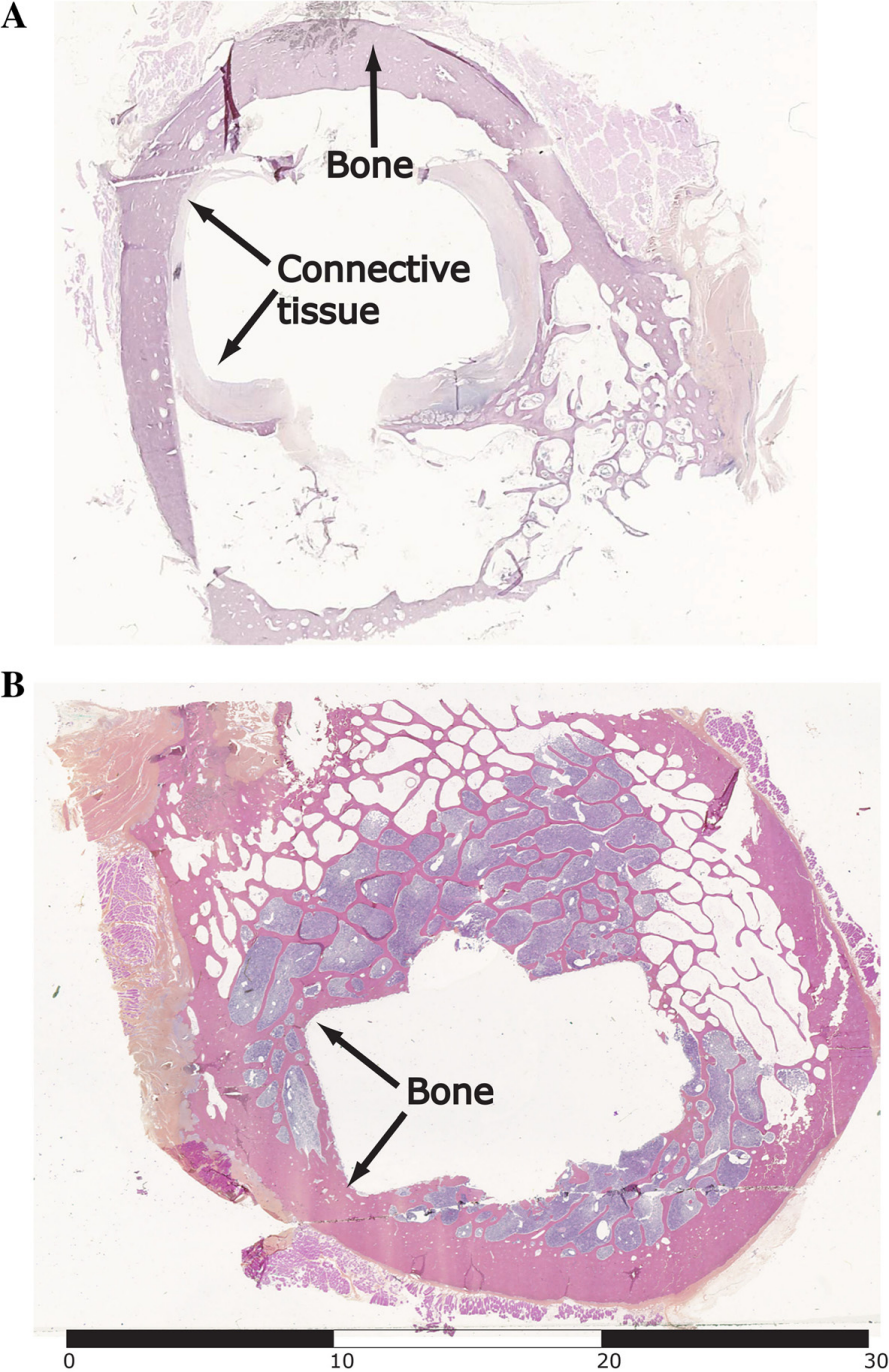
**Histological evaluation of cross-sectioned bone after stem removal.** Microscopic sample from a cross section from the top third of the stem. **(A)** CP group. **(B)** HA group. *Scale* is reported in millimetres.

Microscopic evaluation showed scant bone towards the stem and more connective tissue in the bone/implant interface with calcium phosphate coating, whereas abundant bone formation was seen at the hydroxyapatite (Figure 4). There was no difference in immunoreactive cell response.

## Discussion

The results from this goat model, using titanium femoral stems with different surfaces, demonstrate a higher retention force in favour of hydroxyapatite-coated stems compared with calcium phosphate-coated stems. Of note, the HA-coated stems were only coated in the proximal half, while the CP-coated stems were coated all over. Assessed by microscopy, there was a closer apposition of bone towards the stem in the hydroxyapatite group than in the calcium phosphate group.

Since the introduction of coated, uncemented orthopaedic implants, several coatings have been used to improve their early and long-term fixation [15]. Hydroxyapatite is by far the most commonly used [1]. During the last decade, thinner coatings that are resorbed more quickly have been developed to lower resorption time and reduce the potential for scaling [16],[17]. Thus, there is a reduced foreign body reaction and migration of particles that may cause third-body wear [17]. Calcium phosphate is a thin coating, consisting mainly of brushite, one of the most soluble phases of calcium phosphate, which is electrochemically deposited. Electrochemical deposition can coat geometrically complex structures at room temperature, and active substances such as antibiotics can be added [18]. The thickness can be as low as 20 ± 10 μm and still allows the desired bone ingrowth [19].

To our knowledge, the present large-animal experimental model is unique in that it involves physiologically loaded implants where calcium phosphate coating has been evaluated. In a previously reported rabbit model using unloaded implants, the use of calcium phosphate coating increased bone ongrowth on Ti6Al4V screws when compared with uncoated screws after 3 months of observation [16]. The calcium phosphate coating was similar to the one used in the present investigation. Another study from the same group reported on unloaded calcium phosphate- and hydroxyapatite-coated screws, with observation times of 6, 12 and 52 weeks [20]. Screws with calcium phosphate were coated all over, but those with hydroxyapatite were coated only on one side of the threads due to line-of-sight application of coating. The data showed removal torque force in favour of calcium phosphate, which is in contrast to the results from the present study where the hydroxyapatite-coated implants showed the highest retention force. A further study using a cell culture model showed coverage of newly mineralized calcium phosphate crystals within 48 h with osteoblastic cells, and the authors suggested a better fixation with CP in loaded models than with hydroxyapatite [21]. However, these findings are in contrast to the results in the present study, where physiologically loaded implants were studied after a 6-month period of wear in a large-animal model, with conditions closely mimicking the clinical situation. Indeed, the difference in models may prevent from direct comparison of results.

A femoral stem using coating with calcium phosphate (Bonit®) and TPS (Symax®, Stryker, Montreux, Switzerland) have been used as coating since 2004 [22]. The first study was evaluated by Einzel-Bild-Röntgen-Analyse femoral component analysis (EBRA-FCA; a method of measuring migration of femoral components with two standard radiographs) and showed moderate to low migration patterns in the stems. There have been reports on good bone integration in four recovered stems [23], and there is an ongoing randomized trial between the Symax® stem and another hydroxyapatite-coated stem (Omnifit®, Osteonics Corporation, Allendale, NJ, USA) evaluated by dual-energy X-ray absorptiometry (DEXA) [24]. However, long-term results are not yet available.

In our animal model, we have not analysed the material with EBRA-FCA or DEXA and the low pull-out force is in contrast to the apparent good osteointegration seen in the studies of the Symax® stem. The CP coating was applied on a base of commercially pure porous titanium, not Ti6Al4V as in our model, which can contribute to the good result in the Symax® stem.

Hydroxyapatite is applied to an implant by plasma spraying at a high temperature in the plasma gun, leading to high temperature at the implant (>200°C). This does not permit temperature-sensitive additives in the coating. The coating needs to be thicker but should be less than 80 μm to lower the risk of scaling [17],[25]. In contrast to calcium phosphate, plasma-sprayed hydroxyapatite is applied with the line of sight and cannot reach the inside of porous materials. Hydroxyapatite has longer resorbing time than calcium phosphate, which may lead to foreign body reaction [26]. We found no difference in immunoreactive cell response by histological assessment in the current study.

It appears that hydroxyapatite enhances ingrowth in both stable and unstable conditions [27],[28], whereas calcium phosphate coating is mainly studied with stable implants. The results of the present study support the view that the calcium phosphate coating requires a stable fixation to obtain osteointegration in a loaded situation, compared with hydroxyapatite.

In the calcium phosphate group, the surface coating with commercially pure titanium in the groove had a pore size of 40 μm, but the aim at the design was 40–300 μm. An optimal pore size of above 140 μm has been suggested in other studies [29],[30], in order to allow ingrowth of capillaries and possibly bone. The lack of bone on the calcium phosphate surface might also be attributed to the small pore size.

There are methodological limitations in the present study. The outer geometry of the two stems and the surface of the grooves differ, and as such, they are not immediately comparable. However, the surface area outside the longitudinal groove is comparable, and the groove has the same diameter, which should allow reasonable evaluation of the bone reaction to head-to-head the surface material. The study was not primarily designed to investigate the difference between the coatings, and therefore, there was no power calculation before evaluation. The animals excluded for analyses reduced the number of specimens for evaluation and could have introduced a statistical bias. However, the dropouts from the study from butting among the goats indicate that the animals were subjected to natural behaviour and stresses in the hip joint. The animals did have different post-operative treatments with two separate geographical locations. However, there was no difference in weight and gait score. Thus, the goats seemed to thrive equally well in both facilities.

Our model is used to study implants in loaded conditions, and in future application of the model, there will be improvements with similar experimental conditions, similar implant geometry and possibly uncoated controls when comparing implant coatings.

## Conclusion

Based on the current results of the pull-out strength and microscopic findings, it appears that hydroxyapatite coating of titanium-based stems provides a stronger and more reliable retention of stems than calcium phosphate, as revealed in this animal model involving physiological loading for 6 months. The implications for human applications of the different coatings in hip implants should be further evaluated.

## Competing interests

The authors declare that they have no competing interests.

## Authors’ contributions

KH and ES designed the study and performed the animal studies. KH drafted the manuscript. KH, NRG, ES, KI and KS contributed to the interpretation of results and revision of the manuscript. All authors read and approved the final manuscript.
